# Supplementary material for: Content-rich biological network constructed by mining PubMed abstracts
Source: BMC Bioinformatics. 2004 Oct 8;5:147. doi: 10.1186/1471-2105-5-147 (PMC528731; doi:10.1186/1471-2105-5-147)
Supplement: Additional File 2 — The original results of the above study (non-essential files are deleted to keep the file size under the limit set by BMC bioinformatics). [file 1471-2105-5-147-S2.bz2 › chilibotAdditionalFile2/dip40/343ID8128231E4303/html/IL2RG_IL7.html]

 


 **IL2RG** and **IL7** 
  
Found 129 abstracts in PubMed, retrieved 40.  
 

 What does Google say? 
 PDF only 
| .edu only 

---

**Interactive relationship** (e.g. stimulation, inhibition, etc)

**Neutral relationship**- Canine X linked severe combined immunodeficiency XSCID  [ **IL2RG** ]  is due to mutations in the common gamma gamma c  [ **IL2RG** ]  subunit of the IL 2, IL 4, IL 7  [ **IL7** ] , IL 9 and IL 15 receptors.  Ref: 10507300 Vet Immunol Immunopathol, 1999
- Previously it was known that the IL 2 and IL 7  [ **IL7** ]  receptors shared the common cytokine receptor gamma chain, gamma c  [ **IL2RG** ] , which suggested a possible competition between these cytokines for a receptor component.  Ref: 12354940 Proc Natl Acad Sci U S A, 2002
- Functional forms of the IL 2, IL 4, IL 7  [ **IL7** ] , IL 9, and IL 15 receptors require the gamma c  [ **IL2RG** ]  receptor component.  Ref: 11261789 J Leukoc Biol, 2001
- The IL 7  [ **IL7** ]  receptor is comprised of two chains, IL 7R alpha and gamma c  [ **IL2RG** ] .  Ref: 10379911 Cytokine Growth Factor Rev, 1999
- The high affinity human interleukin 7  [ **IL7** ]  IL 7  [ **IL7** ]  R is a heterodimeric complex consisting of the IL 7Ralpha and common interleukin 2 receptor gamma  [ **IL2RG** ]  IL 2Rgamma c chains.  Ref: 11858939 Cell Signal, 2002
- Interestingly, although Bcl 2 induction was previously suggested to be Jak3 independent, IL 7  [ **IL7** ]  cannot induce Bcl 2 expression in CD4 single positive SP thymocytes in either gamma c  [ **IL2RG** ]  or Jak3 mice nor can IL 7  [ **IL7** ]  rescue CD4 SP thymocytes from dexamethasone induced cell death in gamma c  [ **IL2RG** ]  or Jak3 mice.  Ref: 10653847 Int Immunol, 2000
- In humans, although both T cell and B cell progenitors express the functional IL 7  [ **IL7** ]  receptor that consists of IL 7R alpha and the gamma common gamma c  [ **IL2RG** ]  chain, this lymphocyte receptor system is critical for T lineage but not for B lineage development.  Ref: 11023514 Blood, 2000
- The common cytokine receptor gamma chain gamma c  [ **IL2RG** ]  is an essential receptor component for IL 2, IL 4, IL 7  [ **IL7** ] , IL 9 and IL 15, and thereby gamma c  [ **IL2RG** ]  deficient mice exhibit impaired T cell and B cell development.  Ref: 10653847 Int Immunol, 2000
- The common gamma chain gamma c  [ **IL2RG** ]  is an indispensable subunit of the functional receptor complexes for IL 4, IL 7  [ **IL7** ] , IL 9, and IL 15 as well as IL 2.  Ref: 11418623 J Immunol, 2001
- Canine X linked severe combined immunodeficiency XSCID  [ **IL2RG** ]  is due to mutations in the common gamma gammac subunit of the IL 2, IL 4, IL 7  [ **IL7** ] , IL 9 and IL 15 receptors and has a similar clinical phenotype to human XSCID  [ **IL2RG** ] .  Ref: 10889304 Vet Immunol Immunopathol, 2000
- We show here that naïve T cell survival and memory T cell survival have different requirements for cytokines including the interleukins IL 2, IL 4, IL 7  [ **IL7** ] , IL 9 and IL 15 that use the common cytokine receptor gamma chain gamma c  [ **IL2RG** ] .  Ref: 10881175 Nat Immunol, 2000
- The genetic disorder results from mutations in the common gamma c  [ **IL2RG** ]  chain that participates in several cytokine receptors including the interleukin 2 Il 2, Il 4, Il 7  [ **IL7** ] , Il 9, Il 15 receptors.  Ref: 11265225 Pathol Biol Paris , 2001
- Canine X linked severe combined immunodeficiency XSCID  [ **IL2RG** ]  is due to mutations in the common gamma chain which is a subunit of the receptors of IL 2, IL 4, IL 7  [ **IL7** ] , IL 9 and IL 15.  Ref: 10507301 Vet Immunol Immunopathol, 1999
- The common gamma chain gamma c  [ **IL2RG** ]  that functions both in ligand binding and signal transduction is a shared subunit of the multichain receptors for interleukin IL 2, IL 4, IL 7  [ **IL7** ] , IL 9, IL 15, and IL 21.  Ref: 11815609 J Biol Chem, 2002
- Our laboratory has identified an X linked severe combined immunodeficiency XSCID  [ **IL2RG** ]  in dogs that is the result of mutations in the common gamma chain gammac subunit of the interleukin 2 IL 2, IL 4, IL 7  [ **IL7** ] , IL 9, IL 15, and IL 21 receptors.  Ref: 12857971 Immunol Res, 2003
- Interleukin IL 2, IL 4, IL 7  [ **IL7** ] , IL 9, IL 15, and IL 21 form a family of cytokines based on their sharing the common cytokine receptor gamma chain, gamma c  [ **IL2RG** ] , which is mutated in X linked severe combined immunodeficiency SCID.  Ref: 12435740 J Biol Chem, 2003
- Dendritic cells DCs and DC derived cytokines allowed naive T cells to proliferate selectively in response to IL 4, and potently boosted the response of T CM to IL 7  [ **IL7** ]  and IL 15 by increasing the expression of the IL 2 IL 15Rbeta and the common gamma chain gamma c  [ **IL2RG** ] .  Ref: 11748273 J Exp Med, 2001

**Non-interactive relationship** (e.g. studied together, co-existance, homology, etc.)

- The common cytokine receptor gamma chain gamma c  [ **IL2RG** ] , an essential component of the receptors for IL 2, IL 4, IL 7  [ **IL7** ] , IL 9, and IL 15, is critical for the development and function of lymphocytes.  Ref: 12093291 Biochemistry, 2002
- In the present study, epitope mapping of antagonistic anti gamma c  [ **IL2RG** ]  monoclonal antibodies led to the identification of Asn 128 of mouse gamma c  [ **IL2RG** ]  that represents another potential contact residue that is required for binding IL 2, IL 7  [ **IL7** ] , and IL 15 but not IL 4.  Ref: 11815609 J Biol Chem, 2002
- IL 21R shares the common gamma chain gamma c  [ **IL2RG** ]  with the receptors for IL 2, IL 4, IL 7  [ **IL7** ] , IL 9, and IL 15.  Ref: 12244150 J Immunol, 2002
- IL 4, IL 7  [ **IL7** ] , and IL 15, which do not promote AICD, did not influence FasL or gamma c  [ **IL2RG** ]  expression.  Ref: 10477579 J Immunol, 1999
- We studied the effect of cytokines on reproductive functions such as ovulation and pregnancy, with mutant mice lacking interleukin 2 receptor gamma  [ **IL2RG** ]  IL 2Rgamma, the so called common gamma chain gamma c  [ **IL2RG** ] , which is shared among receptors for multiple cytokines such as IL 2, IL 4, IL 7  [ **IL7** ] , IL 9 and IL 15.  Ref: 12069389 Am J Reprod Immunol, 2002
- IL 7  [ **IL7** ]  signal transduction, involving gamma c  [ **IL2RG** ]  and Jak3, is required for successful recombination at the TCR gamma locus.  Ref: 11404375 J Leukoc Biol, 2001
- In addition to IL 7  [ **IL7** ] , several other cytokines that employ gamma c  [ **IL2RG** ]  as part of their receptor were also capable of stimulating D1 cell survival and proliferation.  Ref: 12609543 J Immunol Methods, 2003
- Among gamma c  [ **IL2RG** ]  related cytokines, interleukin IL 4 and IL 9, but not IL 2, IL 7  [ **IL7** ] , or IL 15, enhanced the proliferation and survival of bone marrow derived mast cells BMMCs from wild type mice.  Ref: 10979963 Blood, 2000
- Sharing of the common IL 10R2 chain between the IL 10 and IL TIF receptor complexes is the first such case for receptor complexes with chains belonging to the class II cytokine receptor family, establishing a novel paradigm for IL 10 related ligands similar to the shared use of the gamma common chain gamma c  [ **IL2RG** ]  by several cytokines, including IL 2, IL 4, IL 7  [ **IL7** ] , IL 9, and IL 15.  Ref: 11035029 J Biol Chem, 2001
- To address whether IL 7  [ **IL7** ]  receptor R activity is essential for early B lineage development in vivo, we examined the frequencies of CLPs and downstream pre pro and pro B cells in adult mice lacking either the alpha chain or the common gamma chain gamma c  [ **IL2RG** ]  of the IL 7R.  Ref: 12208884 J Exp Med, 2002
- The receptor consists of two chains IL 7  [ **IL7** ]  specific alpha chain CD127 and the common gamma c  [ **IL2RG** ]  chain CD132  [ **IL2RG** ]  which is a component of several other cytokine receptors IL 2, IL 4, IL 9 and IL 15.  Ref: 12644318 Immunol Lett, 2003
